# Transmission Quality Classification with Use of Fusion of Neural Network and Genetic Algorithm in Pay&Require Multi-Agent Managed Network

**DOI:** 10.3390/s21124090

**Published:** 2021-06-14

**Authors:** Dariusz Żelasko, Wojciech Książek, Paweł Pławiak

**Affiliations:** 1 Department of Computer Science, Faculty of Computer Science and Telecommunications, Cracow University of Technology, 31-864 Krakow, Poland; wojciech.ksiazek@pk.edu.pl (W.K.); pawel.plawiak@pk.edu.pl (P.P.); 2 Institute of Theoretical and Applied Informatics, Polish Academy of Sciences, 44-100 Gliwice, Poland

**Keywords:** multi-agent managed network, Pay&Require, QoS, wireless networks, machine learning, neural networks

## Abstract

Modern computer systems practically cannot function without a computer network. New concepts of data transmission are emerging, e.g., programmable networks. However, the development of computer networks entails the need for development in one more aspect, i.e., the quality of the data transmission through the network. The data transmission quality can be described using parameters, i.e., delay, bandwidth, packet loss ratio and jitter. On the basis of the obtained values, specialists are able to state how measured parameters impact on the overall quality of the provided service. Unfortunately, for a non-expert user, understanding of these parameters can be too complex. Hence, the problem of translation of the parameters describing the transmission quality appears understandable to the user. This article presents the concept of using Machine Learning (ML) to solve the above-mentioned problem, i.e., a dynamic classification of the measured parameters describing the transmission quality in a certain scale. Thanks to this approach, describing the quality will become less complex and more understandable for the user. To date, some studies have been conducted. Therefore, it was decided to use different approaches, i.e., fusion of a neural network (NN) and a genetic algorithm (GA). GA’s were choosen for the selection of weights replacing the classic gradient descent algorithm. For learning purposes, 100 samples were obtained, each of which was described by four features and the label, which describes the quality. In the reasearch carried out so far, single classifiers and ensemble learning have been used. The current result compared to the previous ones is better. A relatively high quality of the classification was obtained when we have used 10-fold stratified cross-validation, i.e., SEN = 95% (overall accuracy). The incorrect classification was 5/100, which is a better result compared to previous studies.

## 1. Introduction

Nowadays, we can observe a constant increase in the use of computer networks and a dynamic development of techniques used in this type of networks. Currently, networks are one of the foundations of computer systems. It is thanks to computer networks that we obtain the possibility of communication between entities located in any location in the world, and thus it is possible to use any service. Computer networks most often operate in accordance with the best effort principle, i.e., transmission via the operator’s infrastructure takes place without any guarantees as to the quality of transmission. Parameters describing the transmission quality are defined as Quality of Service (QoS) [1,2]. Service, which in this case means the transmission of data over the network. QoS is the answer for users requiring data transmission services meeting specific requirements. Transmission quality can be described by several parameters such as: transmission delay, bandwidth, packet loss ratio, and jitter. It is the values of these parameters that have an impact on the QoS as to which the client has specific expectations. Most often, QoS is obtained by classifying the traffic and prioritizing it [3]. Another solution that was proposed for the purpose of ensuring the quality of the data transmission service is Pay and Require (P&R). The main idea behind P&R is that the customer gets the transmission quality at the expected level. Transmission quality is described using parameters closely related to QoS, i.e., delay, bandwidth, packet loss ratio, and jitter. However, unambiguously describing the influence of the combination of parameters on the overall transmission quality is complicated. Therefore, it was decided to use Machine Learning (ML) for the purposes of finding relationships and converting parameters describing the quality of the data transmission. The proposed solution can be used in networks where the important issue is to ensure transmission quality at the level expected by the customer. It is not easy to assess the quality of the transmission and it can be difficult for the user to understand it (combination of QoS parameters). Therefore, it seems necessary to search for solution that will enable a better understanding of the transmission quality—hence the proposed approach. This type of solution can be very useful in all networks where the important issue is to ensure the quality of transmission at a certain level, and this level is to be understood by the end user. The proposed approach may also be useful for verifying whether the current transmission quality is in line with the customer’s expectations. The current quality measured at a given moment (QoS parameters) in time can be translated to a certain scale using the proposed solution.

ML seems to be more and more popular in different fields, e.g., early detection of hepatocellular carcinoma [4,5], diagnosis of coronary artery disease [6], heart diseases detection [7], tea classification [8], approximation of phenol concentration [9]. There are some works related to ML use in computer networks [10,11], but still, in this field it seems that ML is not very popular. In our research, use of ML still seems to be a novel approach, several studies in this topic were already conducted [10,12,13,14]. In this paper, we proposed an algorithm that allowed us to obtain a better result than the algorithms used so far. Our contributions are as follows:

- Data used in the learning process obtained thanks to the empirical feelings of network users;
- Classification of transmission quality using machine learning;
- An algorithm, which is a fusion of a neural network (NN) and a genetic algorithm (GA) in combination with preprocessing and cross-validation was proposed.

In this study, the same set of input data was used as in the case of previous studies, so that the obtained results could be compared reliably. Data was obtained based on the empirical feelings of test users. A system was developed that allowed the quality of the data transmission service to be assessed. The users were presented with 100 different samples. One sample consisted of a video stream and a website presented to the user with different parameters describing the quality of the data transmission. Each sample consists different set of parameters. The user assessed in the scale of 1–5 the quality of the data transmission service based on the presented materials. Ratings from four different users were obtained in the same way. Then, the obtained results were averaged and it was verified whether there were any excessive differences in assessments—such differences did not occur, so the samples were considered as reliable. To date, several ML-based algorithms have been proposed, with the best obtained classification result of 94%. This article proposes a new algorithm that is a fusion of neural networks (NN) [15,16] and a genetic algorithm (GA). The first mentions of learning NNs can be found as early as 1975 in the works of John H. Holland, one of the authors of GAs [17]. Subsequently, this topic was developed by Whitley [18]. Since then, many scientists have tried to train their NNs with this approach. The use of GA to learn NN can be found in many works. In [19], GA was used to train a multi-layer network to forecast the stock price index. The task of GA was the appropriate selection of weights and biases of the network. The selection of weights with the use of GA has also found application in the nets used for the analysis of time series [20]. The fusion of GA and the NN also works very well in the task of forecasting the weather [21].

Given the popularity of the fusion of NN and GA, it was decided to use this method in the research. The presented approach consists of the following stages: data preprocessing, cross-validation, classification and learning. A better result was obtained than those obtained in the previous research, i.e., overall classification accuracy equal to 95%.

The rest of the paper is organized as follows. State of the art was presented in Section 2. In Section 3, the concept and detailed model were presented. Section 4 presents a description of the experiments. Furthermore, in this section, results were discussed. The paper is completed with Section 5, which includes conclusions and plans for future works.

## 2. Related Work

Quality assurance of data transmission services in computer networks has become a very important issue. Various approaches related to this topic have been proposed over the years. One of the newest is Software-Defined Networking (SDN) [22]. SDN is a centralized solution that is an example of a programmable network [23,24]. The software network allows dynamic configuration and flexible management through the use of open protocols. The SDN separates the logical layer from the physical layer, which is responsible for transmitting data sent over the network. The aim was to obtain a solution that would enable dynamic configuration and modification of the network.

A decentralized solution in which separation of the control and physical layers was proposed as P&R. The main assumption of P&R is to ensure the quality of the service, which is data transmission, in line with customer expectations in IP network (cable or wireless). To achieve this goal, the parameters that describe the quality of the data transmission must be defined. In P&R, the parameters are delay, bandwidth, packet loss ratio and jitter. In order to ensure the quality of the data transmission at the level expected by the customer, it is necessary to constantly monitor parameters and reconfigure the network when necessary. P&R uses an agent system to monitor and reconfigure the network. Agent systems are well known and in some cases used in computer networks [25]. This makes guaranteeing the quality of the data transmission possible. The customer pays a fee for a specific data transmission quality, and the system guarantees continuous availability of the expected resources. The quality of the data transmission was ensured by the use of Policy-Based Routing (PBR). In classical routing, transmission paths are selected based on the transmission destination. In PBR, it is possible to differentiate transmission paths to the same destination depending on the transmission source [26]. Thanks to this approach, packages that originate from different clients with the same destination can travel through different paths. PBR is an example of static routing, i.e., paths are defined in a static manner. P&R uses PBR; however, it uses the agent system to dynamically modify the routing tables. Thanks to this, a dynamic solution was obtained—constantly monitored parameters describing the quality of transmission, and if the transmission path does not meet the customer’s requirements, reconfiguration and change of transmission paths are made. The change of the paths is to adapt the current path (described by a specific transmission quality) to the customer’s requirements. Three types of agents were defined in P&R:

1. Monitoring—one or more instance of such agent appears on every router (physical layer). The task of this agent is to monitor the parameters of individual links. In the first studies, static translation tables of parameters describing the quality (delay, bandwidth, packet loss ratio and jitter) were used—the ranges of individual parameters and the final evaluation of the quality of transmission via a given link were determined. Unfortunately, this solution turned out to be unreliable, so it was decided to use other techniques—first single classifiers and ensemble learning, and in the current work, fusion of NN and GA.
2. Reconfiguration—in a situation where the current transmission parameters do not meet the customer’s requirements, this agent performs the reconfiguration process. The reconfiguration process consists in verifying the quality of transmission through individual paths and selecting the paths in line with customer requirements. Then, the reconfiguration of physical devices is carried out—control commands are sent that change entries in the routing table.
3. Trader—this agent is responsible for carrying out the data transmission quality purchasing process. In P&R, transmission quality is a good that can be purchased for a certain price (e.g., using auctions).

Thanks to the use of agents, a decentralized solution was obtained. Continuous monitoring of parameters and possible network reconfiguration have a positive effect on the quality of the provided data transmission service. The quality of transmission was described using a scale of 1–5, thanks to which this concept is easily understood by customers. The problem was the transformation of parameters that describe the quality (delay, bandwidth, packet loss ratio and jitter) in the adopted scale. One of the proposed solutions is presented in this paper.

**Figure 1:**
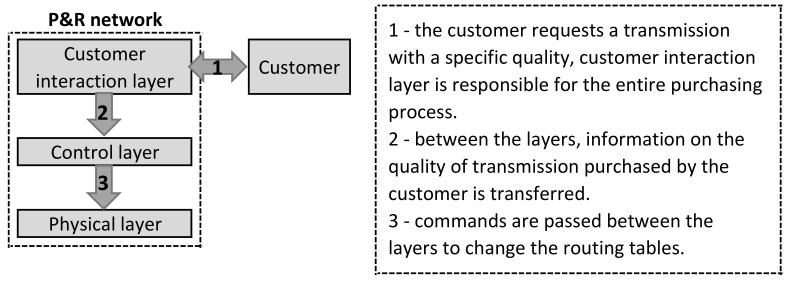
Pay and Require model.

Figure 1 shows the P&R model. There are three layers—physical, control and customer interaction. Network devices operate at the physical layer (IP network—cable or wireless). The control layer is responsible for supervision over the operation of network devices, verification of link parameters and, if necessary, network reconfiguration. The customer interaction layer is used to carry out the data transmission quality purchasing process.

In P&R, it is very important to translate the parameters describing the transmission quality (delay, bandwidth, packet loss ratio and jitter) to a certain scale easily understood by customers. It was decided to use ML, thanks to which the translation takes place in a dynamic and reliable manner. To date, several different classification methods [12] and several ensemble learning algorithms were proposed [14]. The best obtained accuracy was 94%. Therefore, it was decided to propose a new algorithm and conduct relevant research to achieve higher accuracy. Both the algorithm and the research results are presented in this paper. The same set of samples was used to make it possible to compare the obtained results.

## 3. Materials and Methods

### 3.1. Assumptions

- This article proposes a new quality determination algorithm based on the fusion of a NN and GA.
- The samples used at the training stage were obtained from users based on their empirical feelings regarding the quality of the data transmission.
- Thanks to Pay and Require, it is possible to provide data transmission services at the level expected by the customer and this quality is guaranteed.

### 3.2. Dataset

**Figure 2:**
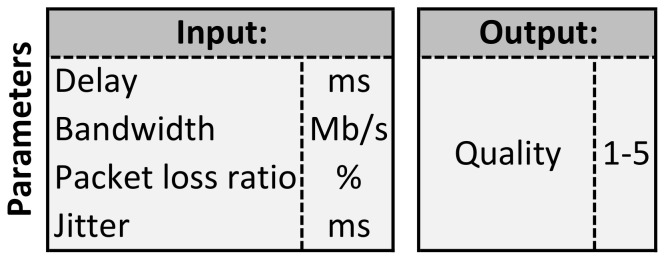
Parameters within sample.

**Figure 3:**
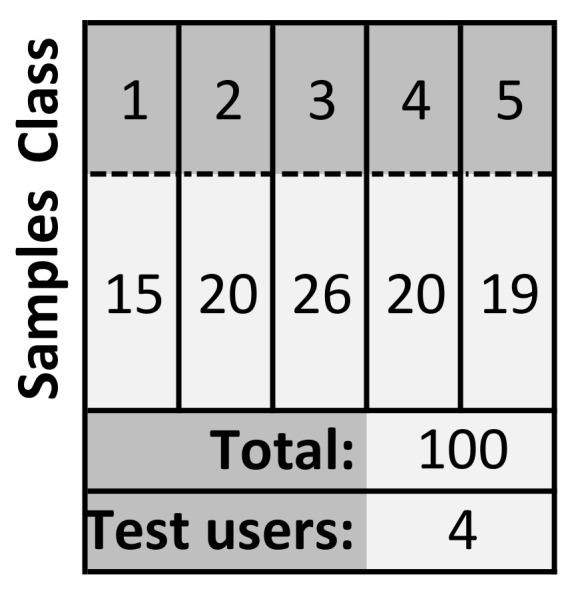
Dataset details.

The samples used in the learning process are a big challenge when using ML. It was decided to use Quality of Experience (QoE). QoE is a technique in which the quality is assessed based on the empirical feelings of the user. By using QoE, the quality is assessed on the basis of the user’s perception. The factors that may influence the user’s experience are, e.g., network devices overload, significant jitter, high packet loss factor. However, the user does not perceive specific parameters, but a specific service and the change in its quality. In order to obtain samples, a system was created in which the test user was presented with two situations—movie streaming and displaying a website. The combination of these two stands for one sample. One hundred different samples were generated. Each sample has different values of parameters describing the transmission quality. These parameters were affected thanks to the use of Trex software (open source network traffic generator) [27] in the test network. The specific parameter values were measured using the iperf program. The user then rated each sample separately in a scale of 1–5. There were four test users, and the results obtained for individual samples were verified for excessive differences (they did not occur) and averaged. Finally one sample is a set of parameters describing the quality and the average rating given by users. These samples were used in the ML process. Details of the samples obtained are presented in Figure 2. Details of the samples in each class are presented in Figure 3. One class stands for one transmission quality grade.

### 3.3. Methods

One of the most difficult tasks to be solved when building an effective machine learning model is the proper selection of its parameters. In most cases, expert knowledge in a given field or experiments carried out earlier is not enough, because each problem is different. When selecting model parameters, scientists mainly rely on similar experiments, the description of which can be found in the literature, or on their previous experiences in other implemented projects. Very often they use the grid search method, which enables them to check a certain pool of solutions. They also try to use randomized search on hyper parameters optimization. However, these approaches are often insufficient. The problem of parameters configuration for machine learning algorithms is often presented as an optimization problem, where the goal is to maximize the accuracy function of a given model. Applied biology inspired algorithms are gaining more and more popularity in optimization problems. In [28], comprehensive learning particle swarm optimizer embedded with local search for higher optimization performance was proposed. In turn, in [29], the authors proposed how to improve production performance under limited labor resources—scheduling problem with limited workers. The authors developed a mathematics model with the objectives of total production cost, makespan, mean flow time and mean idle time of machines in [30].

Genetic algorithms, in particular, have become very popular. These algorithms are successfully used in the case of optimization of machine learning methods, e.g., in the selection of parameters of classifiers enabling the effective classification of hyperspectral images [31], for models used for medical data [4,5,6,32,33] or in the case of financial data [34,35]. Furthermore, papers can be found where other biology-inspired algorithms such as the PSO are used [36,37] or artificial bee colony [38] and for fireworks algorithm [39]. However, genetic algorithms are still the most popular and most effective. We chose this method based on our own experience from previous studies [40,41,42,43]. In our work, we propose the use of genetic algorithms to choose weights of the neural network.

In this article, we propose a new classification method that combines NN and a GA. The quality of the classification was assessed using (a) overall accuracy ratio (SEN—sensitivity [44]) shown in Equation (1), (b) confusion matrices on the test set, and (c) variation of mean square error for various epochs of the GA.

(1) $$SEN=\frac{\sum TP}{\sum (TP+FN)}$$

where:

- *SEN*—sensitivity (overall accuracy),
- *TP*—True Positives,
- *FN*—False Negatives.

**Figure 4:**
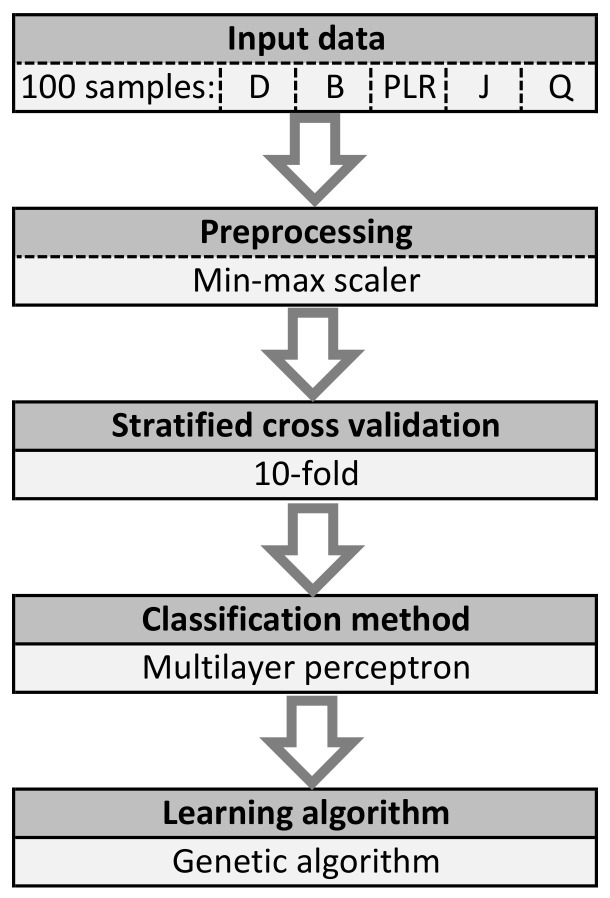
ML algorithm.

The best result is the one for which the highest accuracy and the lowest mean square error were obtained. Proposed algorithm was presented in Figure 4. It was divided into five steps:

1. *Read input data:* The input data consisted of 100 samples. One sample is constructed of delay (D), bandwidth (B), packet loss ratio (PLR), jitter (J) values and the quality (Q) label.
2. *Preprocessing:* One rescaling algorithm was used, i.e., min–max scaler. Thanks to the use of this algorithm, data was obtained in a specific range of values. The presented method uses the scaling to values in the range of 0–1.
3. *Cross validation:* 10-fold stratified cross validation was used. This means that the whole samples set was divided into ten subsets. Nine of these subsets were used at the stage of NN learning and optimizing parameters with the use of a GA. One subset was used to verify accuracy. All studies were performed on test data.
4. *Classification:* For classification purposes, the multilayer perceptron (MLP) was used.
5. *Learning:* To optimize the parameters, GA was used.

In the classical approach, the NN training algorithm is a back propagation error algorithm based on gradient methods.

Artificial neural network (ANN) consists of an input layer, a specified number of hidden layers, and an output layer. ANN are used in many areas, e.g., medicine [45,46], finance [47,48], chemical engineering [49], and computer networks [50,51,52,53]. The basic building block of a neural network is an artificial neuron. In our experiment, we used a standard feed-forward neural network. Input layer neurons are connected to hidden layer neurons, neurons from the hidden layer with neurons from the output layer. Each of these connections has a specific weight—most often in the range from −1 to 1. The purpose of training the neural network is to select weights on the training set in such a way that the network effectively classifies the samples on the test set. The most common method of selecting these weights is the back propagation error method based on gradient methods. Examples of algorithms used are “lbfgs”, “sgd”, “adam”. The mean square error (MSE) is most often used as a measure of network evaluation. MSE can be calculated as Equation (2). Each of these algorithms has additional parameters, such as learning rate, momentum, and the number of learning epochs that must be additionally configured. In addition, the network architecture should be selected, i.e., in particular the number of hidden layers and the number of neurons in the hidden layers. This classic approach does not always achieve good results due to the fact that gradient algorithms are prone to getting stuck in local minima. As a result, the neural network is not able to learn exactly [54].

(2) $$MSE=\frac{1}{2}\sum_{i=1}^{n}{\left(z_{i}-y_{i}\right)}^{2}$$

where:

- *n*—number of the test sets,
- *z_i_*—expected answer for sample *i*,
- *y_i_*—perceptron answer for sample *i*.

In this paper, we propose a method based on the fusion of a NN and a GA. The use of fusion of a NN and a GA was used, e.g., in [7,55,56]. Use of fusion of a NN and a GA is an original solution in the context of data transmission quality classification. There are three ways to link GA and NN together:

1. Selection of the NN architecture through evolutionary computing.
2. Finding the appropriate set of weights of the NN using GA.
3. Combination of the approaches from (1) and (2) [57].

**Figure 5:**
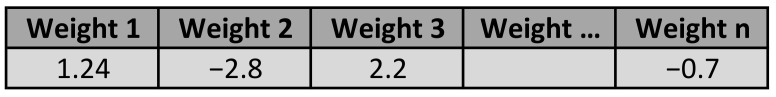
Exemplary individual, *n*—number of optimized weights.

In our research, approach, (2) was used. In the first step, we experimentally selected the architecture of the NN, and then, using evolutionary calculations, we selected the best set of weights. In such a task, an exemplary individual looks like the one depicted in Figure 5.

We chose mean square error (MSE) as the fitness function in the GA, as shown in Equation (2). This approach was used in the literature, for example, to prepare a model of a genetic neural network used to detect heart diseases [7], diagnose breast cancer [58] and for data classification based on DNA [59]. In the literature, other biology-inspired algorithms used to train the NN such as artificial bee colony can be found [60].

### 3.4. Previous Research

To date, several methods of using ML to classify the quality of the data transmission in the P&R network have been proposed. In [12], the authors presented the results for 11 different algorithms. The presented algorithms are available in the Sklearn library. Most of the methods presented are single classifiers, the authors also conducted research for the case of Ensemble Learning. Exactly the same sample set as in this paper was used. The authors verified the classification accuracy (SEN), presented confusion matrices and the best configuration in the case of using individual methods. The confusion matrices allowed to verify in which classes the algorithms were doing better and in which they were worse. In this study, the Stacking Classifier turned out to be the best algorithm, obtaining SEN = 89%.

In the next stage of the research, the authors proposed six new proprietary algorithms based on various classification methods available in the Sklearn library [14]. The proposed algorithms were an example of Ensemble Learning. A different number of classification layers were used with different combinations of classifiers. Of the methods presented, the best result was SEN = 94%. It was decided to conduct research to obtain higher SEN, which is presented in this article.

## 4. Results

This paper proposes an new method of classifying the quality of the data transmission in the P&R network. The main assumption of P&R is to continuously ensure the quality of the data transmission service at the level expected by the customer. This is achieved thanks to the continuous monitoring of parameters describing the quality of the data transmission (delay, bandwidth, packet loss ratio and jitter). The agent environment is responsible for continuous monitoring of parameters. If the current data transmission parameters do not meet the customer’s expectations, the network reconfiguration process is carried out, i.e., the paths through which customer data is sent are modified. It has proven difficult to define static translation tables to describe quality in a certain scale. Therefore, it was decided to use ML. Thanks to the conversion of parameters describing the quality in a certain scale, the values understandable for the client were obtained. The client does not have to understand specific parameters describing the data transmission quality, the information he receives is understandable to them (scale 1–5). This type of parameter conversion is useful for both the client and the agent environment used in P&R network—it is possible to easily describe the quality of the data transmission.

**Figure 6:**
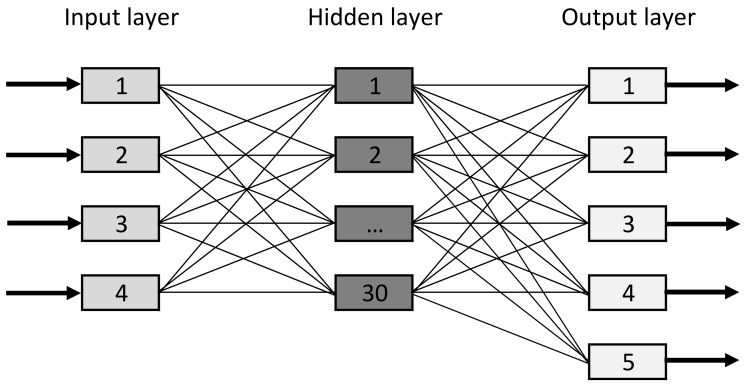
Feed-forward neural network model.

The Python programming language, Sklearn [61] and Deap [62] libraries were used. The results are presented for the test dataset. As part of our experiments, we tested various NN architectures. We focused on modifying the number of neurons in the hidden layer. We tested 5, 10, 15, 20, 25, 30, 40 and 50 neurons in the hidden layer. We obtained the best results when using 30 neurons in the hidden layer. The input layer consisted of four neurons, and the output layer—five neurons (Figure 6). The entire NN required the optimization of 305 parameters (weights and biases). This shows that this is an advanced optimization problem.

Various variants of the configuration of the GA were also tested. In tests, we changed:

- The number of individuals [100, 200, 300, 400, 500, 1000, 2000, 3000];
- Number of epochs [100, 200, 300, 400, 500, 1000, 2000, 3000];
- Type of crossover—two-point, uniform, arithmetic;
- Type of mutation—one, two, three points.

Our best setup was:

- Number of individuals—300;
- Number of epochs—2000;
- Uniform crossover;
- Two-point mutation;
- Elitism—one best individual goes to the next epoch;
- Tournament selection with tournament size 3.

**Figure 7:**
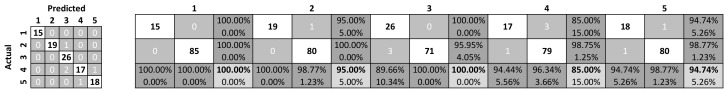
Confusion matrix for the best result (SEN = 95%). Light grey—SEN calculated for individual classes.

We have called our method GA-MLP learning. The best result obtained was SEN = 95%. Figure 7 presents the confusion matrix for the proposed algorithm and the classification results obtained in each class. It can be noticed that in the case of two classes, i.e., 1 and 3, the algorithm achieved 100% SEN. In turn, in class 2, the result was 95%. In class 5, it was slightly less, i.e., 94.74%. The algorithm worked the worst in class 4 with a score of 85%. From the confusion matrix it can be read that the classification errors that occurred always concerned a mistake of +/− one class in relation to the expected value. This means that in the case of data transmission quality classification, the customer may in the worst case get the quality worse or better by 1 than the real one. This situation does not seem to be problematic. The obtained SEN at the moment seems to be satisfactory.

**Figure 8:**
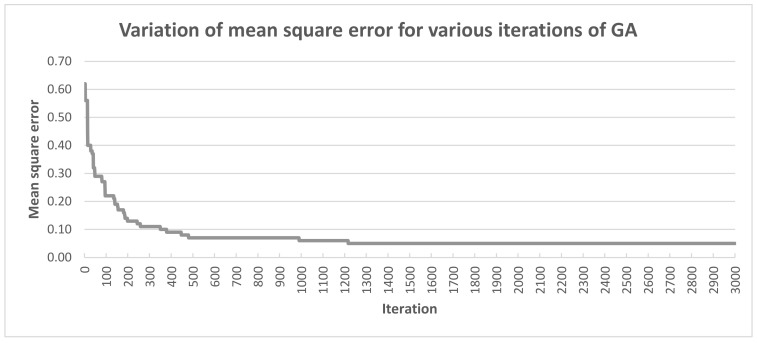
Variation of mean square error for various iterations (epochs) of GA.

Figure 8 shows the convergence chart. A satisfactory relationship can be noticed, i.e., in the following epochs the classification error decreases. It is a very good relationship, proving that the algorithm works correctly both in terms of classification and learning.

**Figure 9:**
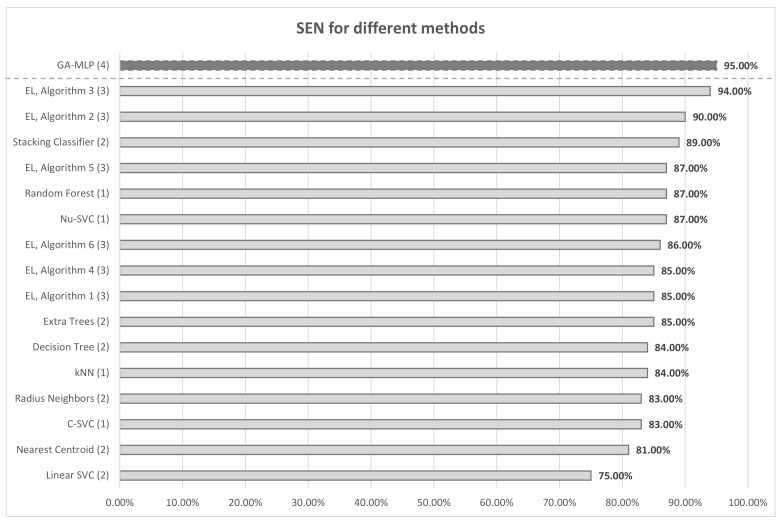
SEN chart for different methods, (1) [12], (2) [13], (3) [14], (4) current research.

**Figure 10:**
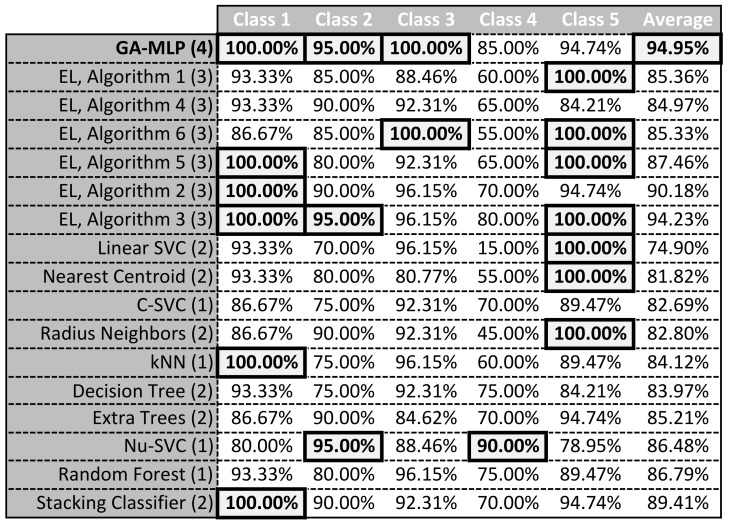
Summary of SEN for individual classes and methods, (1) [12], (2) [13], (3) [14], (4) current research.

Figure 9 and Figure 10 summarize the research carried out so far. Figure 9 is a summary of all SEN values in all tests performed so far—GA-MLP is the result of the research presented in this paper, It can be noticed that the currently proposed method obtained the highest overall SEN. All previously proposed methods, be it Ensemble Learning (EL) or individual classifiers, obtained worse results than the proposed method. This means that taking into account only SEN, the currently presented method is the best. However, in order to get a complete picture of the obtained results, Figure 10 presents the SEN values of individual algorithms in each class. The bold line shows the best results for the different classes. It can be noticed that the proposed algorithm obtained the best results in three classes. In the class that turned out to be the most problematic (considering all the results), i.e., in class 4, the algorithm obtained the second best result. Unfortunately, in class 5, the result could be better. A significant part of the existing algorithms in this class obtained SEN = 100%. Taking into account the results obtained in all classes, it can be stated that the algorithm obtained the best classification result.

## 5. Conclusions

In this paper, we presented:

- The use of a method combining NN and a GA for the classification of the quality of the data transmission in the P&R network;
- Comparison of the results obtained with the method proposed in this article with the results obtained for previous studies, i.e., the use of single classifiers and ensemble learning;
- The method of obtaining samples for the purpose of ML training obtained using QoE;
- The P&R network, the main assumption of which is to provide quality of the data transmission services at the level expected by the customer.

The main problem outlined in this paper is ensuring the quality of the data transmission service. P&R is a solution that allows you to provide data transmission services at the level expected by the customer in IP networks (cable or wireless). This goal was achieved thanks to the use of dynamic differentiation of transmission paths. The dynamics were ensured by the use of agent environment responsible for modifying and reconfiguring transmission paths in a situation where the current quality of the data transmission does not meet the customer’s expectations. It was very important to describe the quality of the data transmission—the most frequently considered parameters are delay, bandwidth, packet loss ratio and jitter. However, understanding the impact of the values of individual parameters on the overall quality of the data transmission turned out to be a significant problem. It is difficult for the end user to understand the above parameters. Therefore, a simple scale was used to describe the quality (1–5). The use of simple scale is advantageous both for end user perception and quality description in an agent-based environment. As a consequence of this approach, the measured parameters describing the quality had to be translated into the adopted scale. It was decided to use ML to solve this problem. To obtain a sufficiently high accuracy of classification, it was necessary to create a set of samples used in the learning process. The samples were obtained from test users based on their empirical feelings about the quality of the data transmission.

The main principle of the proposed solution is its flexibility and reliable translation of parameters describing the transmission quality in a certain scale. Some disadvantages were also noted:

1. The number of samples used in the learning process was 100. This number seems to be sufficient, but using more samples should be considered. A larger number of samples would allow the preparation of a better model—more resistant to overfitting.
2. The reliability of the samples turned out to be very important, because the overall classification accuracy depends on the quality of the samples.
3. The result obtained in the presented research is satisfactory (SEN = 95%), but it seems possible to get an even better result.

Summarizing the presented solution, the obtained result seems to be satisfactory. In subsequent studies, other ML methods can be used to classify the quality of the data transmission. Consideration should also be given to getting more samples from more test users.

## Data Availability

The data presented in this study are available on request from the corresponding author.
